# All that is blood is not schistosomiasis: experiences with reagent strip testing for urogenital schistosomiasis with special consideration to very-low prevalence settings

**DOI:** 10.1186/s13071-015-1165-y

**Published:** 2015-11-10

**Authors:** Stefanie J. Krauth, Helena Greter, Katarina Stete, Jean T. Coulibaly, Seïdinan I. Traoré, Bongo N. R. Ngandolo, Louise Y. Achi, Jakob Zinsstag, Eliézer K. N’Goran, Jürg Utzinger

**Affiliations:** Swiss Tropical and Public Health Institute, Basel, Switzerland; University of Basel, Basel, Switzerland; Centre Suisse de Recherches Scientifiques en Côte d’Ivoire, Abidjan, Côte d’Ivoire; Institut de Recherches en Élevage pour le Développement, N’Djamena, Chad; Center for Infectious Diseases and Travel Medicine, University Hospital Freiburg, Freiburg, Germany; Unité de Formation et de Recherche Biosciences, Université Félix Houphouët-Boigny, Abidjan, Côte d’Ivoire; Laboratoire Régional de Korhogo du Laboratoire National d’Appui au Développement Agricole, Korhogo, Côte d’Ivoire; École de Spécialisation en Élevage de Bingerville, Bingerville, Côte d’Ivoire

**Keywords:** Chad, Côte d’Ivoire, Diagnosis, Microhaematuria, Reagent strip testing, *Schistosoma haematobium*, Schistosomiasis

## Abstract

**Background:**

Reagent strip testing for microhaematuria has long been used for community diagnosis of *Schistosoma haematobium*. Sensitivities and specificities are reasonable, and hence, microhaematuria can serve as a proxy for *S. haematobium* infection. However, assessment of test performance in the context of the underlying *S. haematobium* prevalence is rare and test parameters other than sensitivity and specificity have been neglected.

**Methods:**

Data about the association between microhaematuria and urine filtration results from three studies were compared and put into context with findings from a recent Cochrane review. Data were stratified by *S. haematobium* prevalence to identify prevalence-related differences in test performance. Kappa agreement and regression models were employed to compare data for different *S. haematobium* prevalence categories.

**Results:**

We found a “background” prevalence of microhaematuria (13 %, on average) which does not seem to be associated with schistosomiasis in most settings, irrespective of the prevalence of *S. haematobium*. This background level of microhaematuria might be due to cases missed with urine filtration, or alternative causes apart from *S. haematobium*. Especially in very-low prevalence settings, positive results for microhaematuria likely give an inaccurate picture of the extent of *S. haematobium*, whereas negative results are a sound indicator for the absence of infection.

**Conclusions:**

Reagent strip testing for microhaematuria remains a good proxy for urogenital schistosomiasis, but implications of test results and scope of application differ depending on the setting in which reagent strips are employed. In very-low prevalence settings, microhaematuria is an unstable proxy for urogenital schistosomiasis and treatment decision should not be based on reagent strip test results alone. Our findings underscore the need for highly accurate diagnostic tools for settings targeted for elimination of urogenital schistosomiasis.

## Background

Since the early 1980s, reagent strip testing for microhaematuria has been used as an indirect diagnostic assay for *Schistosoma haematobium* [1, 2]. Indeed, various studies validated reagent strips against standard urine filtration and concluded that the detection of microhaematuria is a valid proxy for urogenital schistosomiasis and related morbidity [3–6]. In a recent Cochrane systematic review it has been summarised that reagent strip testing for *S. haematobium* diagnosis has an overall sensitivity and specificity of 75 % and 87 %, respectively [4]. For specific settings it was suggested that reagent strip testing can be used for individual diagnosis and treatment decision [1, 7, 8], while other groups described reagent strip testing more conservatively as a useful tool for estimating community prevalence [3, 9, 10].

Interestingly, in most settings, there was some proportion of ‘false positive’ (FP) reagent strip test results where microhaematuria could not be associated with *S. haematobium* through reference microscopy [4, 6]. Even after the administration of praziquantel, the prevalence of microhaematuria rarely goes to zero and authors have suggested several explanations for this observation. One suggestion is that some *S. haematobium* infections were missed by microscopy. Especially in low-prevalence settings, *S. haematobium* egg output is generally low and thus hard to be detected by a single filtration of only 10 ml of urine [2, 11]. Repeated urine sampling and use of more sensitive diagnostic assays might remedy this issue [6, 7, 11]. Another explanation is that bladder lesions and associated microhaematuria persisted longer than the actual excretion of eggs into the bladder [12]. A third reason why some microhaematuria is unrelated to urogenital schistosomiasis is that residual menstrual blood or pregnancy in females results in positive reagent strip results [13]. Fourth, it has been noted that tests from different manufacturers performed differently in their ability to detect microhaematuria. For example, the Hemastix® (Bayer Diagnostics; Basingstoke, United Kingdom) proved less sensitive than the Combur9 Test® (Roche Diagnostics; Basel, Switzerland) for microhaematuria and *S. haematobium* infection as confirmed by microscopy [3, 14]. Finally, *S. haematobium* infection is not the only aetiology of microhaematuria [15, 16].

The purpose of this study was to assess the diagnostic accuracy of reagent strips for microhaematuria with particular consideration of settings characterised by low levels of *S. haematobium* prevalence. We addressed three specific research questions. First, does the level of microhaematuria correspond to the level of *S. haematobium* infection in low-prevalence settings? Second, is microhaematuria – that seems unrelated to *S. haematobium –* merely due to missed cases? Third, can microhaematuria be used as a proxy for *S. haematobium* in low-prevalence (<20 %) areas or settings with very-low prevalence that are targeted for elimination (<5 %)?

## Methods

### Ethical considerations

The three study protocols from which original data were obtained for the current analysis (two studies in Côte d’Ivoire, one in Chad) were approved by the institutional research commission of the Swiss Tropical and Public Health Institute (Swiss TPH; Basel, Switzerland) and received clearance from the ethics committees of Basel (EKBB; reference nos. 377/09 and 64/13) and the national ethics committee in Côte d’Ivoire (reference no. 32-MSLS/CNERdkn and 1993 MSHP/CNER). In Chad, research authorization including ethical approval was granted by the ‘Direction Générale des Activités Sanitaires’ in N’Djamena (reference no. 343/MSP/SE/SG/DGAS/2013).

District, regional and local authorities, village chiefs, study participants and parents/guardians of individuals aged below 18 years were informed about the purpose, procedures and potential risks and benefits of the study. Information was provided in the national language (French), as well as common languages spoken in southern and northern Côte d’Ivoire (Baoulé, Dioula/Peulh/Fula and Senoufo) and the Lake Chad area (Arab, Dioula/Peulh/Fula and Kanembou). All authorities and camp/village chiefs were asked for written or oral consent for the conduct of the study in the respective administrative area. In Côte d’Ivoire, written informed consent was obtained from all participants and the parents/guardians of minors. In case of illiteracy, consent was given in front of an impartial witness of the participant’s choosing who signed in the name of the participant. In Chad, informed consent was signed by the camp leader in the presence of an impartial witness after discussion within the group. Due to high illiteracy rates, participating individuals consented orally. These consent procedures had been approved by the respective ethics committees.

Participation was voluntary and there were no further obligations for those who withdrew from the study. All results were coded and treated confidentially. At the end of the studies, all individuals found with a *Schistosoma* infection were offered a single 40 mg/kg oral dose of praziquantel free of charge.

### Data

#### Côte d’Ivoire

During the course of a relatively large study performed in 2014/2015 in the Tchologo region in northern Côte d’Ivoire [17], participants from 28 randomly selected villages, including one to two unofficial settlements in close proximity to the villages, were asked to provide a urine sample. Sample collection was performed throughout the day with 47 % of all samples collected between 10 a.m. and 2 p.m., 83 % before 4 p.m. and 98 % before 6 p.m. Urine samples were transferred to nearby laboratories in Korhogo and Ouangolodougou, where they were subjected to reagent strip testing (Hemastix®, Bayer Diagnostics; Basingstoke, United Kingdom) and the standard urine filtration method. In brief, reagent strips were performed according to the manufacturer’s instructions and results recorded as negative, trace, 1+, 2+ and 3+. With regard to the urine filtration method, one urine sample was examined with a single filtration. In brief, 10 ml of a vigorously shaken specimen were pressed through a 13 mm diameter Nytrel filter with a mesh size of 20 μm (Sefar AG; Heiden, Switzerland), placed on a microscope slide, stained with a drop of Lugol's iodine and then examined under a microscope systematically enumerating *S. haematobium* eggs. All parasitological examinations were performed by the same technician and laboratory assistant. 15 % of the slides were subjected to quality control. In case of discrepancies between the two readings, all slides of the respective day were read a second time.

Additionally, we re-examined data from a study conducted in 2010 in Grand Moutcho in south Côte d’Ivoire that assessed the dynamics of *S. haematobium* egg output following oral administration of a single dose of praziquantel (40 mg/kg). Details of this study have been published elsewhere [18]. In brief, urine samples of two consecutive days were collected from 124 children aged 7–15 years during a baseline survey. Each sample was tested using urine filtration with Lugol’s iodine staining and reagent strip tests (Combur-7-Test®, Roche Diagnostics; Basel, Switzerland) for microhaematuria, proteinuria and leukocyturia. All *S. haematobium*-positive children (*n =* 90) were treated. Subsequently, single urine samples were collected from all treated children on each school day (four times per week) for the first 2 weeks and then twice a week up to 8 weeks post-treatment. All samples were subjected to urine filtration and reagent strip testing for microhaematuria, proteinuria and leukocyturia (Combur 7 Test®).

#### Chad

Urine filtration of 10 ml or whole urine samples from single urine specimens (without Lugol’s iodine staining) and reagent strip testing (Hemastix®, Bayer Diagnostics; Basingstoke, United Kingdom) were likewise performed in the Lake Chad area, where 19 randomly selected groups of mobile pastoralists from four ethnic groups were enrolled in 2013 and 2014. Participants were followed up twice; 6 and 12 months after the baseline survey. Participants found positive for schistosomiasis with urine filtration and/or with a point-of-care cathodic circulating antigen (POC-CCA) urine cassette test for the detection of *Schistosoma mansoni* infection, were treated with a single dose of praziquantel (40 mg/kg). All parasitological tests in Chad were performed on the spot in a mobile laboratory by one of the authors (HG) with assistance from experienced laboratory technicians.

#### Published data from recent Cochrane systematic review

Relevant data from a recent Cochrane systematic review entitled “Circulating antigen tests and urine reagent strips for diagnosis of active schistosomiasis in endemic areas” [4] were extracted and re-organised by prevalence to put our data in the context of the extant literature.

### Statistical analysis

Data were analysed using Stata/IC version 12.1 (StataCorp; College Station, TX, United States of America). A random effects logit regression was employed on our original data with village/camp included as random effect to calculate the relationship between microhaematuria and *S. haematobium* prevalence, the latter confirmed by urine filtration. Reagent strips were read qualitatively (positive or negative). Trace-positive reagent strips were considered as positive. Of note, distinguishing between reagent strip read-out intensities did not change the results notably, except reducing the sample size.

Data from the recent Cochrane systematic review were entered as the number of ‘true positives’ (TP), FP, ‘false negatives’ (FN) and ‘true negatives’ (TN), as reported in the Cochrane systematic review. Subsequent percentages were calculated from these numbers and compared to our data. To examine test performance for different prevalence levels, all baseline and follow-up survey results were grouped according to prevalence categories (0–5 %, 5–10 %, 10–20 %, 20–50 % and 50–100 %).

## Results

### Study participants and prevalence

In the Tchologo region of northern Côte d’Ivoire, 8–33 individuals per village (including nearby Peulh camps) were included in the study. Overall, there were 831 participants and among them, 809 provided a urine sample. Single reagent strip reading and urine filtration were available from 805 and 802 of the participants, respectively, and 801 participants (493 females and 308 males) had complete data. The prevalence of *S. haematobium* based on single urine filtration was 2.2 %, whereas a positive reagent strip test result was noted in 19.5 % of the participants.

In Grand Moutcho, south Côte d’Ivoire, 124 school-aged children (62 females and 62 males) participated in a baseline survey. The prevalence of *S. haematobium* and microhaematuria was 74 % and 62 %, respectively at day 1 of the baseline survey and 70 % and 66 %, respectively at day 2 of the baseline. The overall prevalence of *S. haematobium* and microhaematuria for both baseline days combined was 79 % and 71 %, respectively [18].

**Fig. 1:**
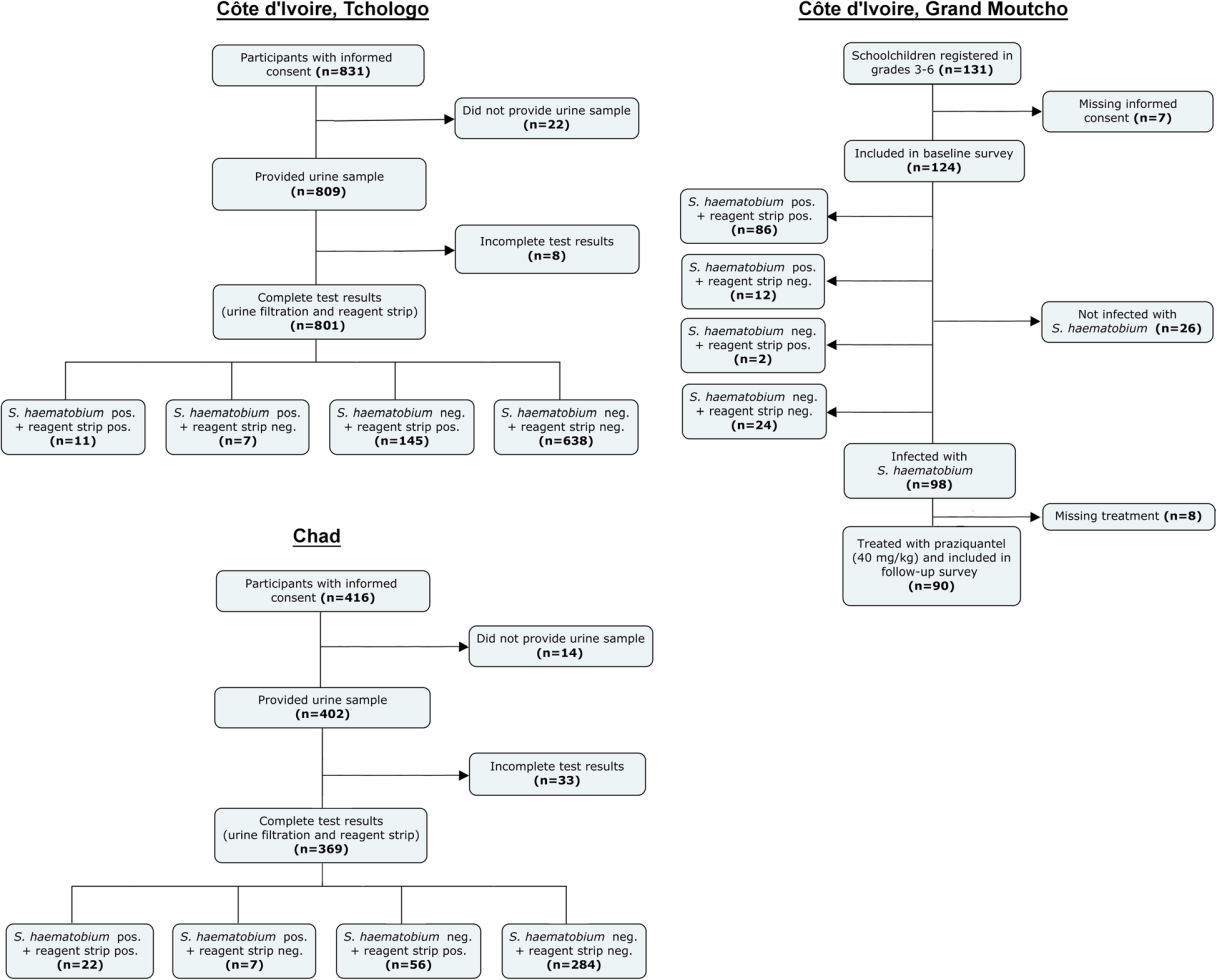
Flowchart of study participation, sample provision and diagnostic tests performed in two sites in Côte d’Ivoire (Tchologo in the northern part and Grand Moutcho in the southern part) and one site in Chad

In Chad, a total of 402 participants provided a urine sample and 369 of them (181 females and 188 males) were tested with reagent strips and urine filtration. 214 individuals (62.9 %) provided a subsequent sample for the first follow-up and 75 (22.1 %) provided a sample for the second follow-up. A total of 60 individuals were treated with praziquantel at baseline as they had a positive test result (either urine filtration or POC-CCA for *S. mansoni*) or because health personnel suggested treatment based on clinical assessment. Urine filtration revealed prevalence of *S. haematobium* of 7.9 % at baseline, 2.7 % at the first and 2.6 % at the second follow-up. Microhaematuria was found in 21.1 % at baseline and in 12.7 % and 10.4 % at the first and second follow-up, respectively (Fig. 1). In all settings, individuals with light intensity infections (egg excretion <50 egg per 10 ml of urine) had a negative reagent strip test result significantly more often (*p <* 0.005) than individuals with heavy infection intensity (egg excretion ≥50 egg per 10 ml of urine).

Of note, the prevalence of *S. mansoni*, as assessed with duplicate Kato-Katz thick smears from a single stool sample in northern Côte d’Ivoire was 0.8 %. In Chad, single Kato-Katz think smears and an ether-concentration method revealed a prevalence of *S. mansoni* of 0.3 %.

### Performance of reagent strip testing compared to urine filtration

In our studies, reagent strip testing resulted in reasonable sensitivities of 61.1 % (north Côte d’Ivoire), 75.9 % (Chad) and 87.8 % (south Côte d’Ivoire). Specificities were somewhat higher; 81.5 % (north Côte d’Ivoire), 83.5 % (Chad) and 92.3 % (south Côte d’Ivoire).

A random effects logit regression between reagent strip tests and urine filtration outcome with village included as random effect, revealed an odds of having a positive filtration when reagent strip testing was positive of 7.4 (95 % confidence interval (CI): 2.3–23.8) in northern Côte d’Ivoire, 86.0 (95 % CI: 18.0–410.8) in southern Côte d’Ivoire and 20.7 (95 % CI: 7.5–57.3) in Chad. The respective Kappa agreements between the filtration results and the reagent strip test results all showed nearly perfect agreement; 0.81 (northern Côte d’Ivoire), 0.88 (southern Côte d’Ivoire) and 0.83 (Chad) [19].

The positive predictive value (PPV), which indicates the likelihood (in %) of being infected with *S. haematobium* if tested positive for microhaematuria, differed greatly from one study to another (7.1 % in north Côte d’Ivoire, 28.2 % in Lake Chad area and 97.7 % in south Côte d’Ivoire). The negative predictive value (NPV; likelihood of not being infected with *S. haematobium* if tested negative with reagent strip), on the other hand, was very high in all of our surveys. A detailed description of test performance in each of the three study sites including the follow-up surveys at Lake Chad are summarised in Table 1. The model predicted odds for having microhaematuria despite a negative urine filtration result at baseline were 7.1 in northern Côte d’Ivoire, 28.2 in the Chad setting and 79.0 in southern Côte d’Ivoire.

**Table 1:** Reagent strip test performance in the three study sites

Study	TP	FP	FN	TN	Egg positive	Microhaematuria	Sensitivity	Specificity	PPV	NPV
Côte d’Ivoire, Tchologo	11	145	7	638	2.2 %	19.5 %	61.1 %	81.5 %	7.1 %	98.9 %
Chad, baseline survey	22	56	7	284	7.9 %	21.1 %	75.9 %	83.5 %	28.2 %	97.6 %
Chad, first follow-up survey	4	24	2	190	2.7 %	12.7 %	66.7 %	88.8 %	14.3 %	99.0 %
Chad, second follow-up survey	1	7	1	68	2.6 %	10.4 %	50.0 %	90.7 %	12.5 %	98.6 %
Côte d’Ivoire, Grand Moutcho	86	2	12	24	79.0 %	71.0 %	87.8 %	92.3 %	97.7 %	66.7 %

*FN* false negative (negative with reagent strip, positive with filtration), *FP* false positive (positive with reagent strip, negative with filtration), *NPV* negative predictive value, *PPV* positive predictive value, *TN* true negative (negative with both, reagent strip and filtration), *TP* true positive (positive with reagent strip and filtration)

### Test performance by prevalence

**Fig. 2:**
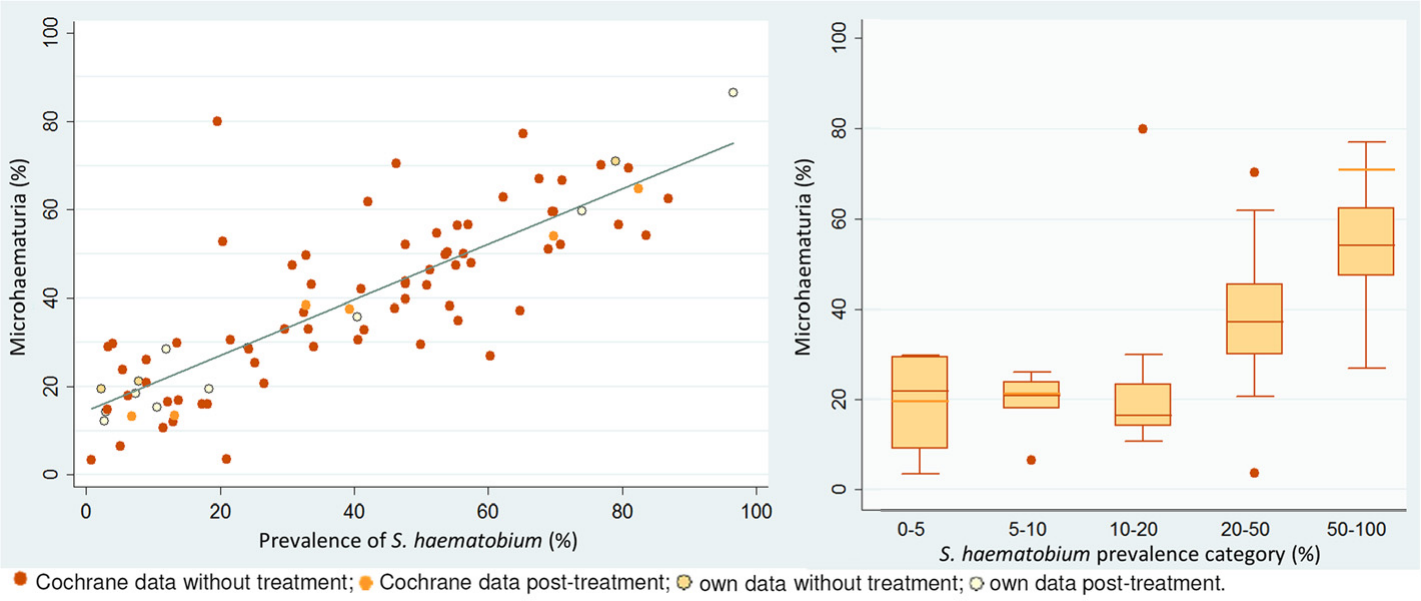
Average microhaematuria over *S. haematobium* prevalence from all surveys as scatter plot and box plot. Light-orange lines in the box plot refer to data from our three surveys (two in Côte d’Ivoire, one in Chad)

Data obtained in our studies and data from the Cochrane systematic review showed that, regardless of the setting, around 15–20 % of the subjects had microhaematuria whenever the prevalence of *S. haematobium* was below 21 %. Above this prevalence level, microhaematuria increased in parallel to *S. haematobium* (Fig. 2).

**Fig. 3:**
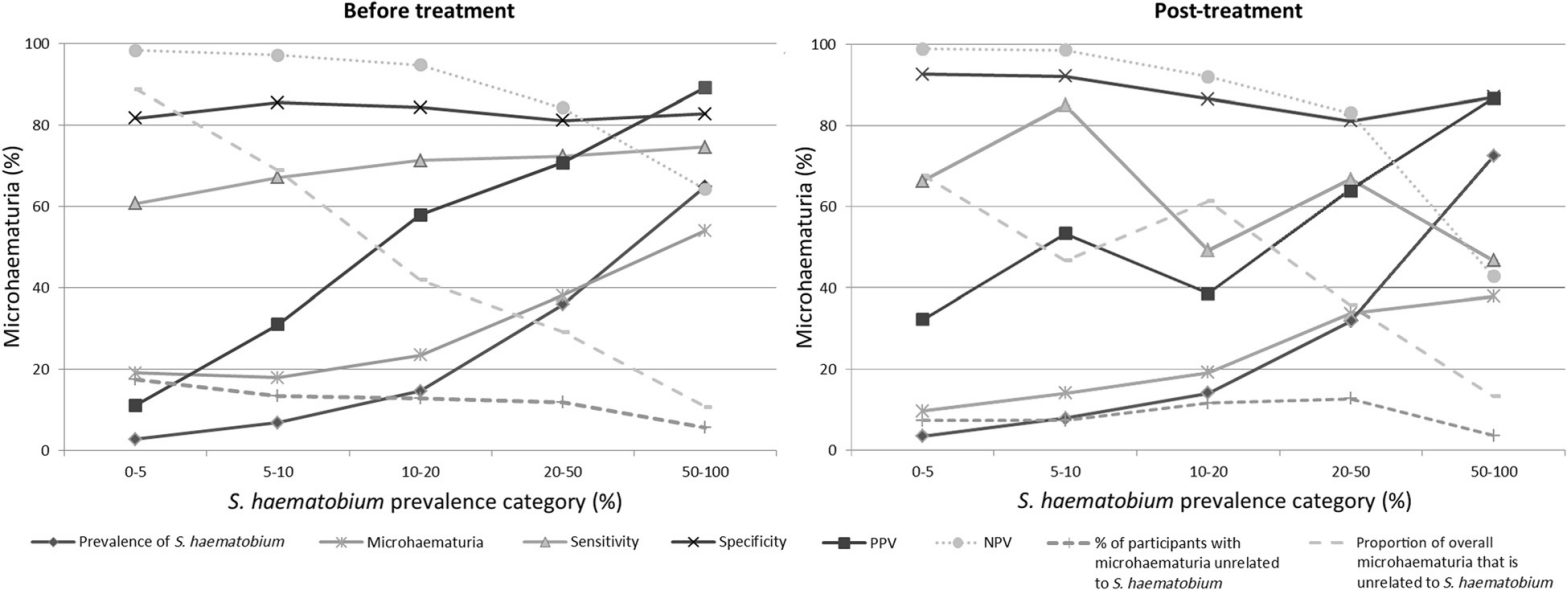
Test parameters (arithmetic mean of all studies) at different prevalence categories before and after treatment. Each follow-up survey in our studies was counted as a separate survey

**Fig. 4:**
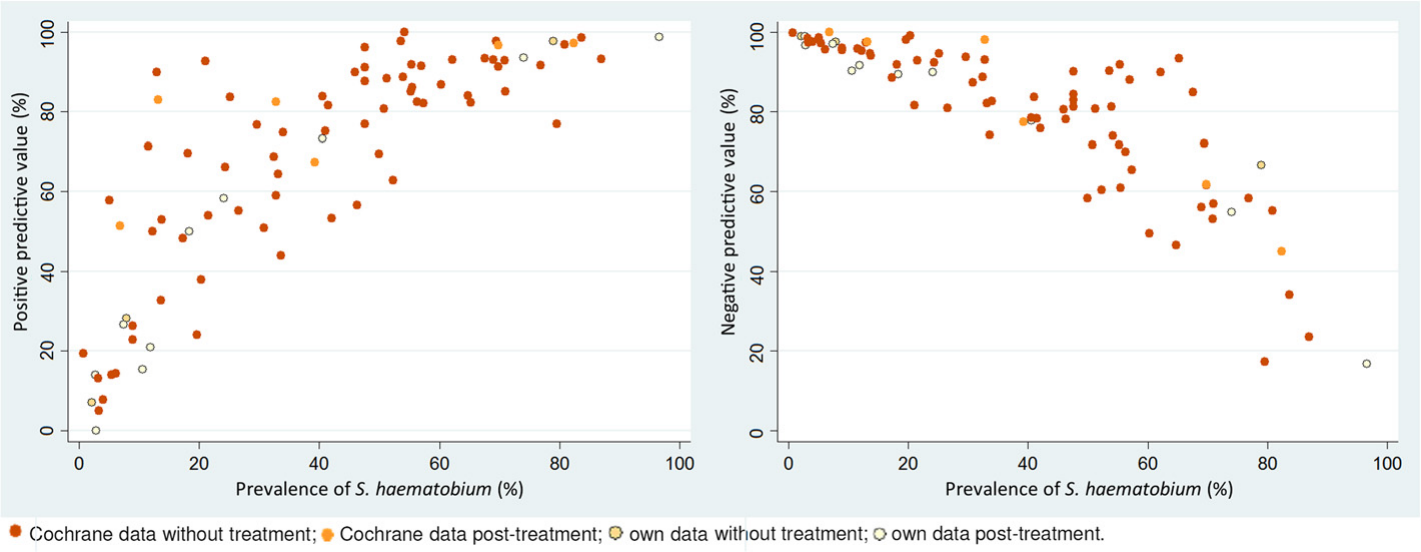
Positive and negative predictive values over *S. haematobium* prevalence.

While sensitivity and specificity were relatively stable over various prevalence levels, PPV and NPV are inherently dependent on prevalence (Figs. 3 and 4). However, the percentage of microhaematuria seemingly unrelated to *S. haematobium* was stable over different prevalence ranges when taken into account that it will be hidden for higher prevalences.

**Fig. 5:**
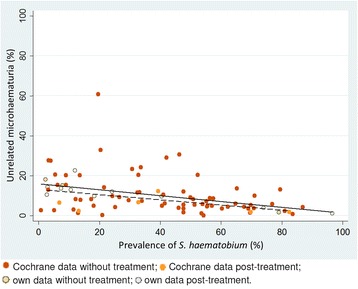
Microhaematuria not associated with *S. haematobium*, stratified by *S. haematobium* prevalence in the study. Solid regression line, all studies; dashed regression line studies post-treatment

**Fig. 6:**
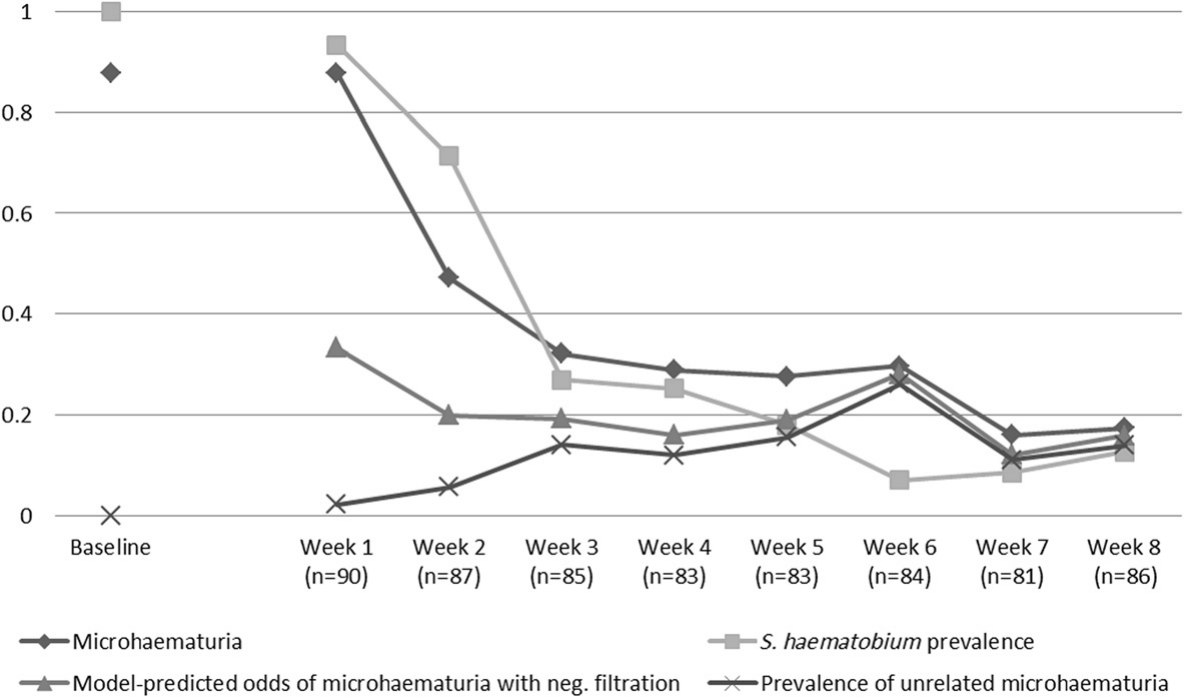
Dynamics of microhaematuria and model-predicted odds of microhaematuria seemingly unrelated to *S. haematobium* over an 8-week period post-treatment of all positive participants

Treatment prior to testing did not substantially alter the picture and seemingly unrelated microhaematuria, although slightly less, showed the same pattern across prevalence ranges (Fig. 5). When the dynamics of microhaematuria was examined over an 8-week period post-treatment for children who tested positive for *S. haematobium* at baseline and were given a single 40 mg/kg oral dose of praziquantel, it was found that, while the prevalence of microhaematuria drops about similarly as the egg output over time, the level of microhaematuria seemingly unrelated to *S. haematobium* egg output quickly reached the same overall background level as found in other studies. The model-predicted odds for having microhaematuria despite a negative filtration result, reached a stable level after the first week post-treatment (Fig. 6).

**Fig. 7:**
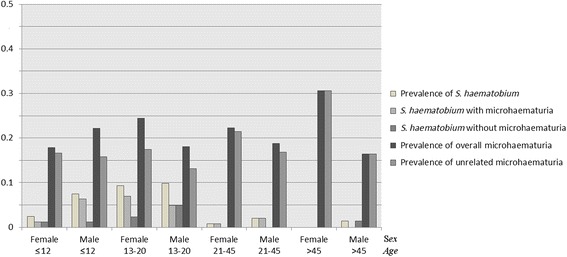
Seemingly unrelated microhaematuria, *S. haematobium* prevalence with and without associated microhaematuria and overall prevalence of microhaematuria by sex and age-group in northern Côte d’Ivoire and in the baseline survey in Chad

Furthermore, our data indicate that this seemingly unrelated microhaematuria is mostly independent of gender. Although females consistently showed slightly higher levels of microhaematuria seemingly unrelated to *S. haematobium* than males in our studies from northern Côte d’Ivoire and Chad, this gender difference was only marginal over all age groups with the exception of females and males aged 45 years and above (Fig. 7).

### Theoretical assessment of the likelihood of missed cases as explanation for seemingly unrelated microhaematuria

If we assume that all cases of “unrelated” microhaematuria can be explained by true *S. haematobium* cases that were missed with urine filtration, the expected prevalence of “unrelated” microhaematuria could be calculated as follows:

Expected true prevalence of *S. haematobium* times the probability to miss a remaining infection.

One way to control for the true prevalence is to consider post-treatment studies only. Yet, although it reduces infection intensity, treatment with praziquantel does not always completely cure an infected individual. Hence, the following assumptions were considered:

- ① 18 % of treated individuals are not fully cured [20];
- ② 20 % of low intensity *S. haematobium* cases are missed by urine filtration (note, the 20 % difference in case-detection stems from a single compared to triplicate urine filtrations in a lightly infected study group) [11]; and
- ③ 5.7 % *S. haematobium* cases present without microhaematuria (note, arithmetic mean of *S. haematobium* infections without microhaematuria from all post-treatment studies from the Cochrane systematic review and our own data, excluding one study of the Cochrane review with an exceptionally high number of *S. haematobium* cases without microhaematuria).

The expected prevalence of FP reagent strips would consist of the uncured *S. haematobium* cases (presenting with microhaematuria) missed by urine filtration. Hence: ① + ② + (1 - ③) = 0.18 * 0.2 * (1–0.057) = 3.4 %. The remaining FP reagent strip results cannot rationally be attributed to missed cases of *S. haematobium*.

If we assume that half of the treatments do not completely cure *S. haematobium* infections, the same rationale would lead to an expected 9.4 % (0.2 * 0.5 * (1–0.057)) of seemingly unrelated microhaematuria which could be explained by missed *S. haematobium* cases. Re-infection can of course increase the prevalence of *S. haematobium* after treatments. The level of re-infection in the included studies is unknown, but it is unlikely to change the numbers to a large extent.

## Discussion

The current comparison of findings of *S. haematobium* eggs in urine and microhaematuria detected by reagent strips confirmed that the usefulness of microhaematuria as a proxy for estimating community prevalence of urogenital schistosomiasis is influenced by the overall prevalence of *S. haematobium* [3, 4, 6, 11, 21, 22]. The Kappa agreement between the two tests revealed very good agreement (>0.80). Moreover, the sensitivity and specificity of microhaematuria have repeatedly been found to be high enough for the reagent strip testing to be a valid diagnostic tool for *S. haematobium* at the community level. These parameters are considerably influenced by the association of *S. haematobium* infection with microhaematuria (94.3 %). However, PPV was very low in low-prevalence settings indicating that either large fractions of microhaematuria in such settings are unrelated to schistosomiasis or that the true prevalence of *S. haematobium* in these settings is grossly underestimated. NPV and PPV are well known to be prevalence-dependent with lower PPV and higher NPV the lower the prevalence of the disease in a given setting [23, 24]. In this sense our findings on the NPV and PPV are not novel, yet, they imply that a positive reagent strip test would not necessarily relate to a positive *S. haematobium* result in very-low prevalence settings.

Our findings have several implications that are offered for discussion. First, regardless of the study settings, there seems to be some level of “background microhaematuria”, which is, at first glance, not directly related to *S. haematobium* infection. The average level of this seemingly unrelated microhaematuria was around 13 % across settings and reported *S. haematobium* prevalences. The observation that background microhaematuria tends to decline with higher prevalence of *S. haematobium* (visualized in Fig. 5) can be explained by the increasing probability to be infected with *S. haematobium*, which will hide any unrelated microhaematuria either because it is being attributed to schistosomiasis or because it co-occurs with *S. haematobium*-induced microhaematuria.

Some authors have argued that most of this background microhaematuria is due to undetected *S. haematobium* explained by the lack of sensitivity of widely used diagnostic tools [25, 26]. However, data from studies performed after praziquantel administration challenge this hypothesis. Indeed, after praziquantel administration, the number of seemingly unrelated microhaematuria attributable to missed *S. haematobium* cases consist of individuals for whom treatment failed to completely clear infection (or perhaps explained by the presence of immature *S. haematobium* flukes that are only marginally affected by praziquantel or the occurrence of rapid reinfection). If 20 % of *S. haematobium* cases are missed by urine filtration [11] and 18 % of positive *S. haematobium* cases treated with praziquantel are not fully cured [20] and 5.7 % of *S. haematobium* infections present without microhaematuria, only 3.4 % (0.18 * 0.2 * (1–0.057)) of seemingly unrelated microhaematuria cases would be attributable to missed cases in post-treatment studies. The remaining unrelated microhaematuria of, say at least 10 %, would remain unexplained and further studies are warranted to assess the cause of this microhaematuria. Potential aetiologies include bladder-stones or urinary tract infections and sickle cell disease as well as persistent bladder lesions from cured *S. haematobium* infections [15, 27–29]. Truly FP reagent strip results have also been reported, and are thought to be caused by the presence of semen in urine [30]. However, some studies suggest that cure rates of *S. haematobium* infections after praziquantel administration vary greatly depending on the study and the diagnostic effort and can be as low as 50 % [31, 32] which would indicate that the majority of the background microhaematuria that we found throughout settings would indeed be explained by *S. haematobium* infections missed by urine filtration. If this assessment holds, it would mean that there is a persisting *S. haematobium* prevalence of around 10–15 % in settings which were characterised to have lower prevalences. In turn, this would change the current picture about schistosomiasis burden of disease as well as the evaluation of the success of schistosomiasis control and elimination programmes. Indeed, a recent study on a promising high-sensitivity diagnostic tool based on the detection of a circulating anodic antigen (CAA) did find a prevalence of 13.3 % of *S. haematobium* with the antigen test when urine filtration found a prevalence of only 3.3 % and reagent strip testing found a prevalence of 4.1 % [33].

Due to the lack of a ‘gold’ standard in *S. haematobium* diagnosis, it cannot be ascertained 100 % whether this background haematuria results from missed *S. haematobium* cases or from alternative causes of microhaematuria. The level of seemingly unrelated microhaematuria did not differ much between males and females for different age groups, excluding the explanation of unrelated microhaematuria caused by pregnancy or menstruation. If the observed background haematuria is due to different aetiologies, such as bladder-stones or urinary tract infections [15, 27, 28], it follows that individual treatment-decisions targeting *S. haematobium* should not merely be based on reagent strip results, particularly in low-prevalence settings.

In either case, researchers, health care personnel and disease control managers need to be aware that in settings with *S. haematobium* prevalence below 20 %, especially in settings targeted for elimination, a positive reagent strip test should always be followed up with urine filtration or better with other, more sensitive, diagnostic assays. Moreover, in view of the likelihood to miss an infection, which is, in addition, higher in low-intensity infections, and the fact that egg output shows a considerable day-to-day fluctuation [3, 34, 35], the follow-up diagnosis should contain multiple samples over consecutive days.

Taken together, while reagent strip testing remains a valid tool for rapid assessment of community prevalence of *S. haematobium* [3, 9, 10] or even for individual diagnosis [2, 4, 7], one has to consider the epidemiological setting in which the test is executed as well as the goals of a schistosomiasis control and elimination programme or the specific research questions.

Praziquantel is a safe and efficacious drug that is recommended for preventive chemotherapy against schistosomiasis [36, 37]. As preventive chemotherapy is escalating and schistosomiasis elimination is the new goal [38, 39], settings characterised by very-low prevalence and intensity of *Schistosoma* infection are becoming the norm rather than the exception and this has important ramifications for the use of diagnostic assays.

## Conclusion

Irrespective of the true cause of the persisting background prevalence of microhaematuria—be it missed *S. haematobium* cases or alternate causes for microhaematuria—the overwhelming implication of our findings and those of other researchers, is that there is a pressing need for more accurate diagnostic tools (higher sensitivity and higher specificity) if we indeed want to aim for elimination of schistosomiasis in selected settings [26, 33, 36, 38]. New research and funding efforts should target the well-known weaknesses of currently available diagnostic assays for urogenital schistosomiasis.
